# Impacts of High Fructose Diet and Chronic Exercise on Nitric Oxide Synthase and Oxidative Stress in Rat Kidney

**DOI:** 10.3390/nu15102322

**Published:** 2023-05-16

**Authors:** Gaizun Hu, Lusi Xu, Osamu Ito

**Affiliations:** 1 Department of Molecular Physiology and Biological Physics, Center for Membrane and Cell Physiology, School of Medicine, University of Virginia, Charlottesville, VA 22903, USA; nvf2ec@virginia.edu; 2 Department of Internal Medicine and Rehabilitation Science, Tohoku University Graduate School of Medicine, Sendai 983-8536, Japan; 3 Division of General Medicine and Rehabilitation, Faculty of Medicine, Tohoku Medical and Pharmaceutical University, Sendai 983-8536, Japan

**Keywords:** fructose, exercise, nitric oxide system, oxidative stress, kidney

## Abstract

Chronic exercise (Ex) exerts antihypertensive and renoprotective effects in rats fed a high fructose diet (HFr). To elucidate the mechanisms, the impacts of an HFr and Ex on the nitric oxide (NO) system and oxidative stress in the kidney were examined. Rats were fed a control diet or an HFr, and a part of the HFr-fed rats underwent treadmill running for 12 weeks. The HFr did not affect nitrate/nitrite (NO_x_) levels in plasma and urine, and Ex increased the NO_x_ levels. The HFr increased thiobarbituric acid reactive substance (TBARS) levels in plasma and urine, and Ex decreased the HFr-increased TBARS levels in plasma. The HFr increased the neuronal and endothelial NO synthase (nNOS and eNOS) expressions, and Ex enhanced the HFr-increased eNOS expression. The HFr inhibited the eNOS phosphorylation at serine 1177, and Ex restored the HFr-inhibited eNOS phosphorylation. The HFr increased xanthine oxidase and NADPH oxidase activities, and Ex restored the HFr-increased xanthine oxidase activity but enhanced the HFr-increased NADPH oxidase activity. The HFr increased the nitrotyrosine levels, and Ex attenuated the HFr-increased levels. These results indicate that although Ex enhances the HFr-increased eNOS expression and NADPH oxidase activity, an HFr inhibits renal eNOS phosphorylation and NO bioavailability, whereas Ex ameliorates them.

## 1. Introduction

Fructose is a monosaccharide sugar that is presented in fruits and vegetables. It has been widely used in manufactured food products in Western countries since the middle of the 20th century. Consuming sugar is important for the epidemics of lipid disorders, abdominal obesity, and type 2 diabetes [1,2,3]. Numerous studies have shown a strong relationship between a high fructose diet (HFr) and the etiology of metabolic syndrome [4,5]. Fructose consumption has been increasing for decades and plays a critical role in the development of hypertension and metabolic syndrome. An HFr may also have a relationship with chronic kidney disease (CKD) since metabolic syndrome is a significant cause for CKD [6]. Clinical trials have reported that consuming sugar-sweetened beverages is related with hypertension among healthy subjects [7] and patients with CKD [8]. In CKD patients, the low fructose diet lowered blood pressure [9]. In previous studies, we examined the impact of an HFr on renal damage in rodents. A 60% fructose diet induced renal interstitial fibrosis, podocyte injury, and glomerular sclerosis in male Sprague Dawley rats [10].

The renal impacts of fructose have been reported. HFr upregulates sodium and chloride transporters, resulting in salt absorption, and induces salt-sensitive hypertension [11]. HFrs also affect the renal nitric oxide (NO) system. An HFr decreases the renal expression of endothelial NO synthase (eNOS) protein caused by the dietary sodium load in a high-salt diet (3% NaCl) [12], and an HFr decreases the renal expression of eNOS mRNA [13] and urinary nitrate/nitrite (NO_x_) excretion [13,14]. Additionally, the gene transfection of eNOS increases the eNOS expression and urinary NO_x_ excretion and decreases blood pressure in HFr-fed rats [14].

Fructose has also been reported to induce oxidative stress in the kidney. An HFr increases nicotinamide adenine dinucleotide phosphate (NADPH) oxidase activity and thiobarbituric acid reactive substance (TBARS) levels, which is an index of oxidative stress-induced lipid peroxidation in rat kidney [15]. However, an HFr does not induce the increase in the renal NADPH oxidase activity during dietary sodium restriction with a low salt diet (<0.01% NaCl) [16]. The NADPH oxidase inhibitor apocynin prevents blood pressure elevation, the aortic production of superoxide anion, and impaired acerylcholine-induced relaxation in HFr-fed rats [17]. Fructose at 1 mmol/L, which is a physiological concentration, increases the production of superoxide anions in human proximal tubular cells, and the xanthine oxidase (XO) inhibitor oxypurinol partially reduces the fructose-induced production of superoxide anions. [18]. The selective XO inhibitor febuxostat lowers blood pressure elevation and reduces renal vasoconstriction and glomerular pressure in HFr-fed rats [19]. In HFr-fed rats, both the NO precursor L-arginine and the superoxide dismutase (SOD) mimetic tempol decrease blood pressure with increases in the renal medullary blood flow and urinary sodium excretion [20], and the antioxidant flavonoid (-)-epicatechin decreases blood pressure and increases renal NOS activity [17]. These results suggest that there is an interaction between the NO system and oxidative stress, which regulates renal hemodynamics, salt absorption, and blood pressure in HFr-fed rats.

Chronic exercise (Ex) has been shown to exert antihypertensive and renoprotective effects in animals and humans with hypertension and CKD [21]. Ex with voluntary wheel running attenuates the blood pressure elevation in HFr-fed rats [22]. We previously reported that Ex with treadmill running exerts antihypertensive and renoprotective effects in HFr-fed rats [10]. Ex increases the renal eNOS and neuronal NOS (nNOS) expressions, the NOS activity, and the urinary NO_x_ excretion in spontaneously hypertensive rats (SHRs), Wistar Kyoto rats (WKYs), and Zucker diabetic rats [23,24]. Ex also ameliorates oxidative stress by decreasing the NADPH oxidase activity in SHRs [22], the XO activity in Dahl salt-sensitive (DS) rats [25], and both NADPH oxidase activity and XO activity in rats with 5/6 nephrectomy [26]. However, the impacts of Ex on the renal NO system and oxidative stress have not been reported in HFr-fed rats. We hypothesized that Ex might restore the HFr-induced changes in the renal NO system and oxidative stress. Therefore, this study examined the impacts of an HFr and Ex on the renal NO system and oxidative stress.

## 2. Materials and Methods

### 2.1. Animals and Protocol

Male Sprague Dawley rats (Charles River Laboratories, Yokohama, Japan) at 5 wk of age were used. The rats were housed in an animal care facility with a controlled temperature of 24 °C under a 12:12 h light-dark cycle with food and water ad libitum. The protocol was carried out after a one-week acclimation. All involved rats were reviewed and received prior approval by the Animal Welfare Committee of Tohoku University.

After the acclimation, the rats were divided into 3 groups (*n* = 8 in each group). The sample size calculations are provided by G Power 3.1. The projected total sample size needed with power = 0.80 is approximately *n* = 21. Thus, our proposed total sample size of N = 24 will be adequate for the objective of this study. The standard lab chow pellets were replaced by a control diet (TD. 05075, Envigo Teklad Diets, Madison, WI, USA) or an HFr (60% fructose, TD. 89247), which induced metabolic syndromes as previously reported [10]. The control diet contained 46% carbohydrates, which is mainly composed of starch, whereas the HFr contained 60% fructose as the carbohydrate. The caloric content of both diets was 3.6 kcal/g. The percentage of fat, protein, carbohydrate, and NaCl content are equivalent in Con and HFr. The sedentary groups received either the control diet (CON-Sed) or HFr (HFr-Sed), and the exercise group received HFr and underwent Ex (HFr-Ex) with treadmill (KN-73, Tread-Mill; Natsume Industries Co., Tokyo, Japan) [10,23,24,25,26]. Initially, the animals started at 10 m/min, 0°, and 10 min/day, after which the treadmill speed was increased from 10 to 20 m/min progressively and to 60 min/day in 1 week. After acclimation, the rats underwent treadmill running for the remaining 11 weeks, which was maintained at an aerobic intensity (20 m/min, 0°, 60 min/day, and 5 days/week) [10].

### 2.2. Blood Pressure Measurement

The systolic blood pressure (SBP) was measured every 2 weeks from the start of Ex using a tail-cuff monitor (CODA-HT4, Kent Scientific, Torrington, CT, USA) described previously [23]. The rats were pre-heated on a thermostatic heater, and the temperature of the tail was measured. After the tail’s temperature reached 32 °C, the rats were kept still for at least 20 min, and 25 consecutive circles of measurement were performed in a quiet environment. The lower accepted readings were recorded as the SBP under still conditions.

### 2.3. Sample Preparation

The urine samples of rats kept in metabolic cages were collected on ice for 24 h and stored at −80 °C for further analysis. The rats were returned to their cages after the 12th week exercise and kept still for 48 h prior to the sacrifice. After being anesthetized with pentobarbital (i.p., 50 mg/kg BW), the plasma samples were collected via decapitation. Urine and plasma samples were centrifuged at 2000 g for 15 min; the supernatant was collected for further analysis. To evaluate renal function, urinary albumin and plasma creatinine were measured (Oriental Yeast, Nagahama, Japan; SRL, Tokyo, Japan). The kidneys were quickly removed, and the cortex was homogenized in a buffer described previously [10]. The protein concentration was measured using the Bradford method.

### 2.4. Measurement of NO_x_ and TBARS

The NO_x_ and TBARS levels in plasma and urine were measured by commercial kits (NO_x_, Nitrate/Nitrite Colormetric Assay Kit, 780001; TBARS, TBARS Assay Kit, 10009055, Cayman, MI, USA) described previously [23]. Data are expressed as nmol/mL for plasma NO_x_ and TBARS levels and nmol/day for urinary NO_x_ and TBARS excretion.

### 2.5. Western Blot Analysis

Western blot analyses were performed to elucidate the protein expression and phosphorylation as described previously [27]. Renal cortical homogenates (60 μg) were loaded onto SDS-polyacrylamide gels and separated electrophoretically. Proteins were transferred to membranes and incubated with primary antibodies raised against nNOS, eNOS, and phosphorylated (p-) eNOS at serine 1177 and threonine 495 (nNOS, 610308, RRID: AB_397700; eNOS, 610297, RRID: AB_397691; p-eNOS^S1177^, 612392, RRID: AB_399750; p-eNOS^T495^, 612706, RRID: AB_399946; BD Biosciences, USA); Akt and p-Akt at serine 473; AMP-kinase (AMPK) and p-AMPK at threonine 172 (Akt, 9272S, RRID: AB_329827; p-Akt^S473^, 9271S, RRID: AB_329825; AMPK, 2532S, AB_330331; p-AMPK^T172^, RRID: AB_331250; Cell Signaling Technology, MA, USA); and XO and nitrotyrosine (XO, sc-20991, RRID: AB_2214858; nitrotyrosine, sc-32757, RRID: AB_628022; Santa Cruz Biotechnology, TX, USA). The intensities of the bands (at 140 kDa for eNOS and p-eNOS, at 155 kDa for nNOS, at 60 kDa for Akt and p-Akt, at 64 kDa for AMPK and p-AMPK, and at 70 kDa for nitorotyrosine) were quantified using Image J software (Ver.2.0.0; National Institutes of Health, Bethesda, MD, USA) and normalized to those of the reference protein β-actin at 40 kDa, and the mean intensity of bands for the CON-Sed group was assigned a value of 1. The phosphorylated proteins were represented as the ratio of phosphorylated proteins to their total protein expressions.

### 2.6. NADPH Oxidase and XO Activities

The activity of NADPH oxidase and XO were measured by lucigenin-enhanced chemiluminescence and pterin-based fluorescence assay, as described previously [23,28]. For the NADPH oxidase activity assay, the samples were co-incubated with lucigenin, and background luminescence was recorded. After the addition of NADPH, the activated luminescence was measured for 5 min, and the luminescence change in counts per minute (C.P.M) was recorded. For the XO activity assay, the samples were incubated with pterin at 37 °C for 120 min after the background fluorescence record. The fluorescence changes in C.P.M were represented as the XO activity.

### 2.7. Statistical Analysis

Data are presented as means ± SE. The data were analyzed using two-way repeated-measures ANOVA or one-way ANOVA, followed by a Tukey test for multiple comparisons among the groups. *p* < 0.05 was considered to indicate statistical significance. Data were analyzed by GraphPad Prism (version 9, GraphPad, La Jolla, CA, USA).

## 3. Results

### 3.1. Impacts of HFr and Ex on SBP, Albuminuria, and Plasma Creatinine

**Figure 1:**
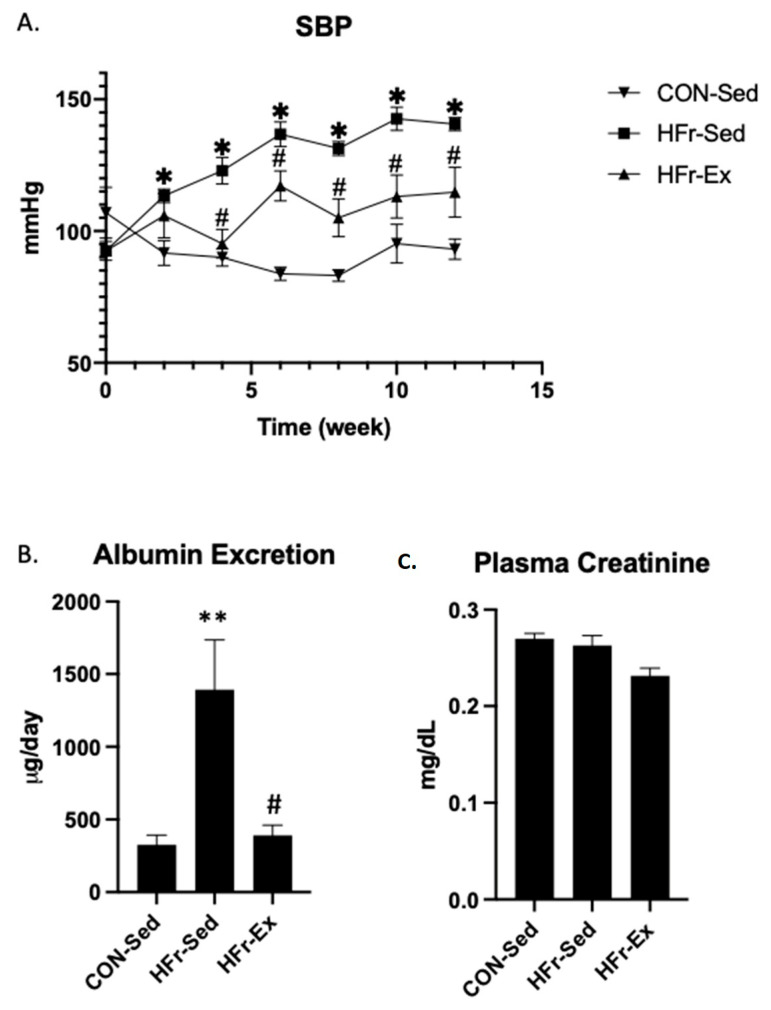
Impacts of high fructose diet (HFr) and chronic exercise (Ex) on systolic blood pressure (SBP), albuminuria, and plasma creatinine. Male rats were divided into 3 groups, and the sedentary groups were fed either a control diet (CON-Sed) or a high fructose diet (HFr-Sed), while the exercise group received HFr and underwent treadmill running (HFr-Ex) for 12 weeks. (**A**) The time course of SBP in the CON-Sed (black down-pointing triangle), HFr-Sed (black square), and HFr-Ex (black up-pointing triangle) groups. (**B**) Urinary albumin excretion and (**C**) plasma creatinine were compared among the CON-Sed, HFr-Sed, and HFr–Ex groups. Data are presented as means ± SE for *n* = 8 rats per group. * *p* < 0.05, ** *p* < 0.01 compared to the CON-Sed group; # *p* < 0.05 compared to the HFr-Sed group.

The HFr significantly increased systolic blood pressure, and Ex significantly inhibited the HFr-induced elevation (Figure 1A). The HFr significantly increased albuminuria and plasma creatinine, and Ex significantly inhibited the HFr-increased albuminuria and plasma creatinine (Figure 1B,C).

### 3.2. Impacts of HFr and Ex on NO_x_ and TBARS in Plasma and Urine

**Figure 2:**
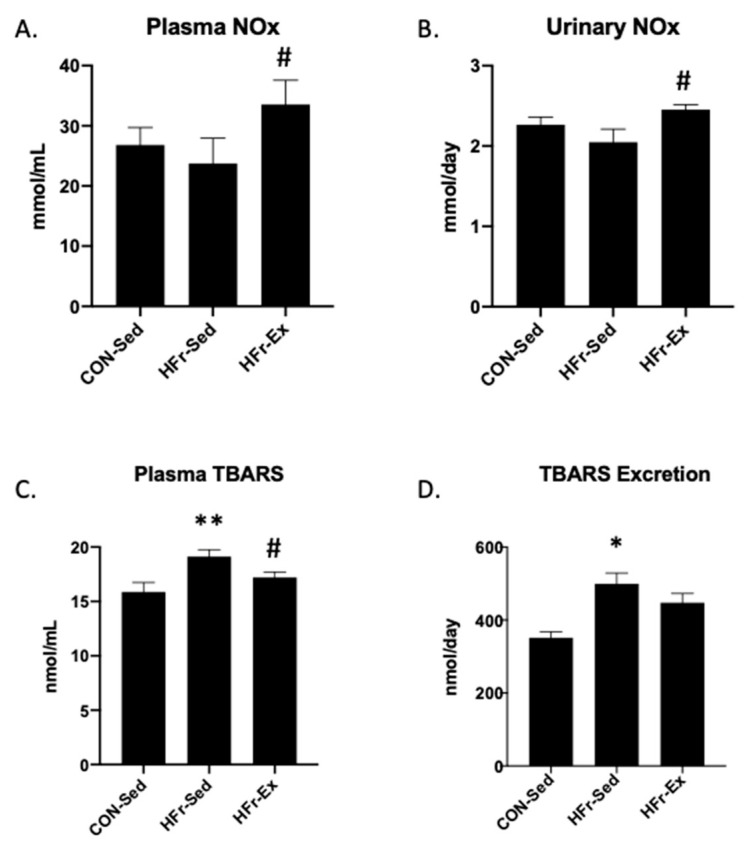
Impacts of HFr and Ex on the nitrate/nitrite (NO_x_) and thiobarbituric acid reactive substance (TBARS) levels in plasma and urine. (**A**) Plasma NO_x_ concentration; (**B**) urinary NO_x_ excretion; (**C**) plasma TBARS concentration; (**D**) urinary TBARS excretion were compared among the CON-Sed, HFr-Sed, and HFr–Ex groups. Data are presented as means ± SE for *n* = 8 rats per group. * *p* < 0.05, ** *p* < 0.01 compared to the CON-Sed group; # *p* < 0.05 compared to the HFr-Sed group.

The HFr did not affect the NO_x_ levels in plasma or urine, and Ex significantly increased the NO_x_ levels in plasma and urine (Figure 2A,B). The HFr significantly increased the TBARS levels in plasma and urine, and Ex inhibited the HFr-increased levels in plasma but not in urine (Figure 2C,D).

### 3.3. Impacts of HFr and Ex on NOS Expression and Phosphorylation

**Figure 3:**
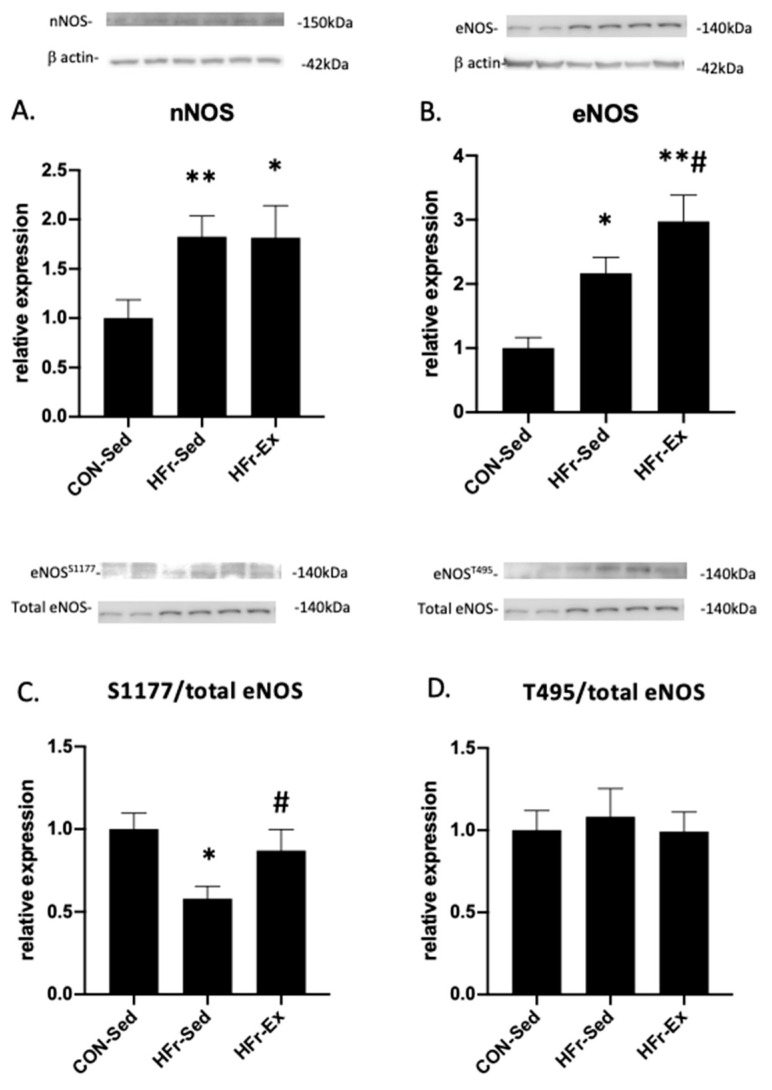
Impacts of HFr and Ex on nitric oxide synthase (NOS) expression and phosphorylation. (**A**) Relative expression of total neuronal NOS (nNOS); (**B**) relative expression of total endothelial NOS (eNOS); (**C**) quantitative ratios of phosphorylated (p−) eNOS at serine 1177 to total eNOS; (**D**) quantitative ratios of p−eNOS at threonine 495 to total eNOS compared among the CON-Sed, HFr-Sed, and HFr–Ex groups. Data are presented as means ± SE for *n* = 8 rats per group. * *p* < 0.05 and ** *p* < 0.01 compared to the CON-Sed group; # *p* < 0.05 compared to the HFr-Sed group; **# *p* < 0.01 compared to the CON-Sed group and *p* < 0.05 compared to the HFr-Sed group.

The HFr significantly increased the nNOS expression, and Ex did not affect the HFr-increased expression (Figure 3A). The HFr significantly increased the eNOS expression, and Ex enhanced the HFr-increased expression (Figure 3B). The HFr significantly inhibited the eNOS phosphorylation at serine 1177, and Ex restored the HFr-inhibited eNOS phosphorylation to the control levels (Figure 3C). The HFr and Ex did not affect the eNOS phosphorylation at threonine 495 (Figure 3D).

### 3.4. Impacts of HFr and Ex on Akt and AMPK Expression and Phosphorylation

**Figure 4:**
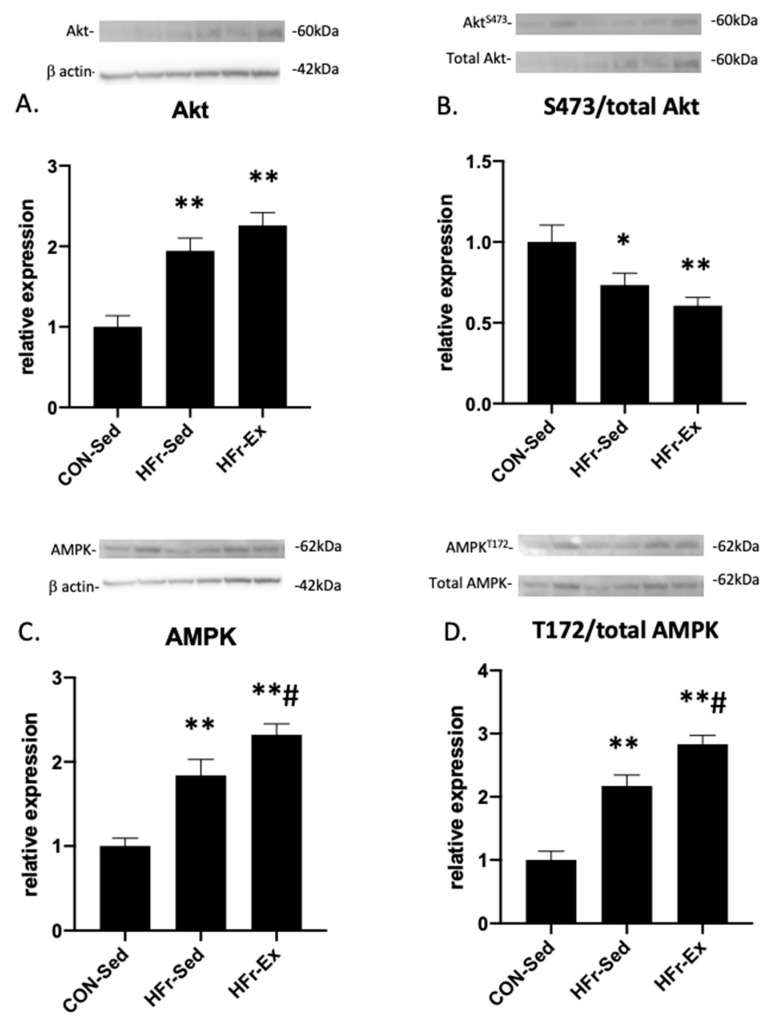
Impacts of HFr and Ex on Akt and AMP−kinase (AMPK) expression and phosphorylation. (**A**) Relative expression of total Akt; (**B**) quantitative ratios of phosphorylated Akt at serine 473 to total Akt; (**C**) relative expression of total AMPK; (**D**) quantitative ratios of phosphorylated AMPK at threonine 172 to total AMPK compared among the CON-Sed, HFr-Sed, and HFr–Ex groups. Data are presented as means ± SE for *n* = 8 rats per group. * *p* < 0.05 and ** *p* < 0.01 compared to the CON-Sed group; **# *p* < 0.01 compared to the CON-Sed group and *p* < 0.05 compared to the HFr-Sed group.

The HFr significantly increased the total Akt expression, and Ex did not affect the HFr-increased total Akt expression (Figure 4A). The HFr and Ex did not affect the Akt phosphorylation at serine 473 (Figure 4B). The HFr significantly increased the total AMPK expression, and Ex enhanced the HFr-increased total AMPK expression (Figure 4C). HFr significantly increased the AMPK phosphorylation at threonine 172, and Ex enhanced the HFr-increased AMPK phosphorylation (Figure 4D).

### 3.5. Impacts of HFr and Ex on NADPH Oxidase and XO Activity and Nitrotyrosin Levels

**Figure 5:**
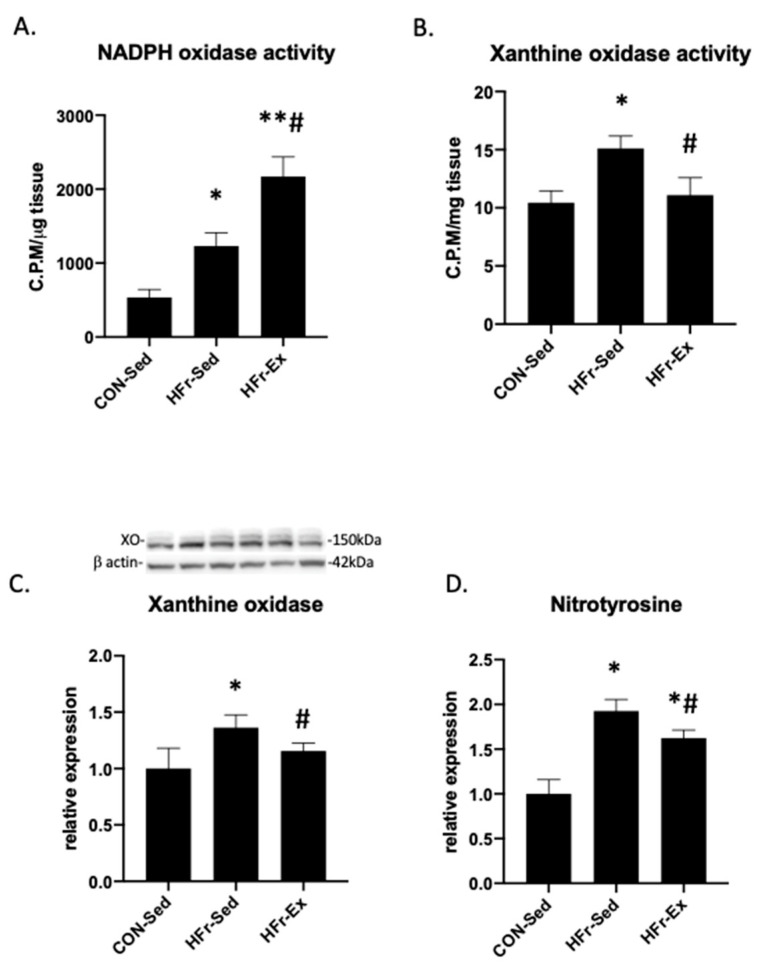
Impacts of HFr and Ex on the nicotinamide adenine dinucleotide phosphate (NADPH) oxidase activity, the xanthine oxidase activity and expression, and the levels of nitrotyrosine. (**A**) NADPH oxidase activity; (**B**) xanthine oxidase activity; (**C**) relative expression of xanthine oxidase; (**D**) nitrotyrosine levels compared among the CON-Sed, HFr-Sed, and HFr–Ex groups. Data are presented as means ± SE for *n* = 8 rats per group. * *p* < 0.05 compared to the CON-Sed group; # *p* < 0.05 compared to the HFr-Sed group; *# *p* < 0.01 compared to the CON-Sed group and *p* < 0.05 compared to the HFr-Sed group; **# *p* < 0.01 compared to the CON-Sed group and *p* < 0.05 com-pared to the HFr-Sed group.

The HFr significantly increased the NADPH oxidase activity, and Ex enhanced the HFr-increased NADPH oxidase activity (Figure 5A). The HFr significantly increased the XO activity, and Ex restored the HFr-increased XO activity to the control levels (Figure 5B). The HFr significantly increased the XO expression, and Ex restored the HFr-increased XO expression to the control levels (Figure 5C). The HFr significantly increased the renal nitrotyrosine levels, which is an indicator of ONOO^−^ formation, and Ex attenuated the HFr-increased nitrotyrosine levels (Figure 5D).

## 4. Discussion

Ex exerts antihypertensive and renoprotective effects in HFr-fed rats [10]. This study examined the impacts of an HFr and Ex on the renal NO system and oxidative stress. The HFr did not affect NO_x_ levels, and Ex increased the levels in plasma and urine. The HFr increased the nNOS and eNOS expressions, and Ex enhanced the HFr-increased eNOS expression. The HFr inhibited the eNOS phosphorylation at serine 1177, and Ex restored the HFr-inhibited eNOS phosphorylation. The HFr increased the NADPH oxidase and XO activities, and Ex restored the HFr-increased XO activity but enhanced the HFr-increased NADPH oxidase activity. The HFr increased the renal nitrotyrosine levels, and Ex attenuated the HFr-increased nitrotyrosine levels. To our knowledge, this study is the first to report the impacts of Ex on the renal NO system and oxidative stress in HFr-fed rats.

Previous studies have reported the impact of an HFr on renal NOS expression in HFr-fed rats [12,13,15]. Nishimoto et al. reported that an HFr (40% fructose) for 2 weeks decreased the eNOS expression in the renal medulla during high-salt diet (3% NaCl) intake, although the HFr did not affect the urinary NO_x_ excretion [12]. Gordish et al. reported that an HFr (20% fructose) for 2 weeks reduced the urinary NO_x_ excretion caused by a high-salt diet (4% NaCl) by 40% [29]. Hasegawa et al. reported that an HFr (60% fructose) for 6 weeks decreased the renal eNOS mRNA expression and the urinary NO_x_ excretion in rats [13]. Prince et al. reported that an HFr (10% fructose in drinking water) for 8 weeks decreased the renal nNOS protein expression but did not affect the renal eNOS protein expression [15]. In contrast to the previous studies, the HFr for 12 weeks increased the renal eNOS and nNOS expressions. The discrepancy in the impacts of an HFr on the NOS expression in the studies might be explained in part by the feeding protocol and the period of the HFrs. Regarding the time-dependent impact of fructose on the eNOS expression, fructose reduced the NO release from human aortic endothelial cells; however, the eNOS protein expression decreased at 4 h and returned to the control levels after 8 h [30]. Ex enhanced the HFr-increased eNOS expression but did not affect the HFr-increased nNOS expression. We previously reported that Ex increased the renal eNOS and nNOS expressions in SHRs, WKYs, and Zucker diabetic rats [23,24].

NO production is substantially adjusted by NOS activity, which is regulated by fluid shear stress and numerous agonists via cellular events, including increases in intracellular Ca^2+^ and the interaction with substrate and co-factors, as well as adaptor and regulatory proteins and and protein phosphorylation. The eNOS phosphorylation of serine 1177 enhances activity, whereas the phosphorylation at threonine 495 inhibits activity [31]. The HFr did not increase the urinary NO_x_ excretion in spite of the increase in the renal eNOS and nNOS expressions, suggesting an inhibition of eNOS activity by the HFr. Therefore, this study also examined the renal eNOS phosphorylation and shows that the HFr inhibited the eNOS phosphorylation at serine 1177 and that Ex restored the HFr-inhibited eNOS phosphorylation. In contrast to the present results, Galleano et al. reported that an HFr (10% fructose in drinking water) for 8 weeks stimulated the NOS activity and the eNOS phosphorylation at serine 1177 in the kidney and aorta [15,32]. Mederious et al. reported that HFr (10% fructose in drinking water) decreased the eNOS expression without changes in the eNOS phosphorylation at serine 1177 and that Ex restored the HFr-decreased eNOS expression and stimulated the eNOS phosphorylation in the aorta [33]. Stanisic et al. reported that HFr (10% fructose in drinking water) inhibited the eNOS phosphorylation at serine 1177 and that Ex restored the HFr-inhibited eNOS phosphorylation in the heart [34].

The phosphorylation of eNOS at serine 1177 is mediated by Akt, AMPK, protein kinase A, and Ca^2+^/calmodulin-dependent protein kinase II [31]. This study further examined the renal Akt and AMPK expression and phosphorylation and shows that the HFr increased the Akt expression without changes in the Akt phosphorylation at serine 473 and that Ex did not affect the HFr-increased Akt expression or the phosphorylation. The HFr significantly increased the AMPK expression and the AMPK phosphorylation at threonine 172, and Ex enhanced the HFr-increased AMPK expression and AMPK phosphorylation. In contrast to the present results, Korkmaz et al. reported that an HFr (20% fructose in drinking water) for 15 weeks decreased the renal Akt and eNOS expressions [35], and Gu et al. reported that an HFr (10% fructose in drinking water) for 12 weeks inhibited the Akt phosphorylation at serine 473 in the kidney [36]. Fructose inhibited the Akt phosphorylation at serine 473 in human proximal tubular cells [36]. Wahba et al. reported that an HFr (10% fructose in drinking water) for 12 weeks inhibited the renal AMPK phosphorylation at threonine 172 during high-salt diet (3% NaCl) intake [37].

This study shows that the HFr increased the renal NADPH oxidase activity and that Ex enhanced the HFr-increased NADPH oxidase activity. In agreement with the present results, an HFr (10% fructose in drinking water) for 8 weeks increased the renal NADPH oxidase activity [15], and fructose increased the XO-dependent production of superoxide anions in human proximal tubular cells [18]. We have previously reported the impacts of Ex on NADPH oxidase and XO in several hypertensive and kidney disease models [23,25,26]. Ex decreased the NADPH oxidase activity in SHRs, whereas Ex increased the NADPH oxidase activity in WKYs [23]. Ex also decreased the XO activity in DS rats [25] and both NADPH oxidase and XO activities in rats with 5/6 nephrectomy [26]. These results suggest that Ex might increase the NADPH oxidase in normotensive rats and that Ex might decrease the XO activity in rats with salt-sensitive hypertension as well as HFr-fed rats.

In a previous study, Price et al. also examined the impacts of an HFr on antioxidant enzymes, including SOD, glutathione, and catalase, in the kidney [15]. HFr increased the activity and expression of the cytosolic SOD isoform (CuZn SOD) but did not affect the activity or expression of the mitochondrial SOD isoform (Mn SOD), glutathione peroxidase, or catalase. Additionally, dietary supplementation with the antioxidant flavonoid (-)-epicatechin decreased renal oxidative stress, TBARS, and nitrotyrosine levels in the HFr-fed rats, but it did not affect significantly the activity or expression of CuZn SOD. These results suggest that the impacts of an HFr and (-)-epicatechin might reflect an adaptive mechanism against renal oxidative stress [15]. We previously examined the impact of Ex on renal SOD activity in normotensive WKYs and SHRs, in which renal oxidative stress and NOS expressions are higher like in HFr-fed rats, and Ex did not affect renal SOD activity in WKYs or SHRs [38].

This study had several limitations. First, we examined impacts of an HFr and Ex on only the renal NADPH oxidase and XO as the source of superoxide anions. Exercise has been reported to increase the production of reactive oxygen species and result in oxidative stress in numerous tissues [39,40]. We also reported that Ex stimulates the mitochondrial fatty acid β-oxidation in the renal cortex [19]. Additionally, local hypoxia and energy depletion during exercise were reported to increase oxidative stress [39,40]. Second, we examined impacts of an HFr and Ex on only Akt and AMPK as the mediators of the eNOS phosphorylation at Ser 1177. Because HFr and Ex affect blood pressure, renal hemodynamics, and the endocrine metabolic system, including plasma insulin levels, it is possible that the impacts of an HFr and Ex on the eNOS phosphorylation at Ser 1177 might be modulated complexly by Akt, AMPK, protein kinase A, and Ca^2+^/calmodulin-dependent protein kinase II. Third, we did not fully clarify the interaction between the renal NO system and oxidative stress in the renal eNOS and nNOS expression. Fructose induces the eNOS uncoupling via oxidative stress and inhibits eNOS activity [31]. In endothelial cells, hydrogen peroxide increases the eNOS expression and phosphorylation at Ser 1177 [41,42]. We previously reported that NADPH oxidase-derived hydrogen peroxide increases the renal NOS expressions and NO production in SHRs and WKYs [43] and enhances the Ex-increased renal NOS expressions and NO production in SHRs and WKYs [38]. A further study is necessary to examine the impacts of Ex on eNOS uncoupling and the effect of NADPH oxidase and XO inhibitors on the renal NO system in HFr-fed rats.

In conclusion, an HFr inhibits the renal eNOS phosphorylation at Ser 1177 in spite of the increases in eNOS and nNOS expressions, and Ex enhances HFr-increased eNOS expression and restores HFr-inhibited eNOS phosphorylation, suggesting that Ex increases renal NO production. Ex enhances the HFr-increased NADPH oxidase activity, whereas Ex restores HFr-increased XO activity and attenuates renal nitrotyrosine levels. Ex exerts antihypertensive and renoprotective effects probably by increasing renal NO production and NO bioavailability in HFr-fed rats.

## Data Availability

The data presented in this study are available on request from the corresponding author. The data are not publicly available due to private reason.
